# A review of the genus *Apronopa* van Achterberg (Hymenoptera, Braconidae, Alysiinae) with a key to species

**DOI:** 10.3897/zookeys.793.29313

**Published:** 2018-10-29

**Authors:** Francisco Javier Peris-Felipo, Sergey A. Belokobylskij

**Affiliations:** 1 Bleichestrasse 15, Basel CH-4058, Switzerland Unaffiliated Basel Switzerland; 2 Zoological Institute, Russian Academy of Sciences, St Petersburg, 199034, Russia; Museum and Institute of Zoology, Polish Academy of Sciences, Wilcza 64, Warszawa 00-679, Poland Zoological Institute, Russian Academy of Sciences St Petersburg Russia

**Keywords:** Braconidae, Alysiinae, *
Apronopa
*, parasitoid wasp, Palaearctic, redescription, illustration, key

## Abstract

A review of the genus *Apronopa* van Achterberg, 1980 with illustrated re-descriptions of the known species and a key for their identification is provided.

## Introduction

The genus *Apronopa* van Achterberg, 1980 is small, with only three known species, and rarely collected taxon with a Palaearctic distribution (Yu et al. 2016). This genus is a distinct member of the *Aspilota* generic-group. The main characters for diagnosis of *Apronopa* are *Aspilota*-like wing venation (with very narrow and long pterostigma), absence of dorsope on the first metasomal tergite and usually a sculptured basal part of second metasomal tergite (van Achterberg 1980, Belokobylskij 1998).

The main aim of this study is the preparation of a complete and richly illustrated redescription of all known *Apronopa* species (some of which have non-English original descriptions), estimation of their diagnostic species characters and preparation of the first comprehensive key for determination of all described species.

## Materials and methods

For the terminology of morphological features, sculpture and measurements see Peris-Felipo et al. (2014); for wing venation nomenclature see van Achterberg (1993); for measurements of the marginal cell see Peris-Felipo and Belokobylskij (2017). Material was imaged using a Digital Microscope Keyence VHX-2000 and Adobe Photoshop^®^ imaging system. The types of described species are deposited in the collection of the Hungarian Natural History Museum (Budapest, Hungary; HNHM), the National Museum of Natural History (Leiden, the Netherlands; RMNH), the Zoological Institute of the Russian Academy of Sciences (St Petersburg, Russia; ZISP) and the Zoological Institute of the Russian Academy of Sciences (München, Germany; ZISP).

## Taxonomic results

### Class Hexapoda Blainville, 1816

#### Order Hymenoptera Linnaeus, 1758

##### Family Braconidae Nees, 1811

###### Subfamily Alysiinae Leach, 1815

####### Tribe Alysiini Leach, 1815

######## Genus Apronopa

Taxon classificationAnimaliaHymenopteraBraconidae

van Achterberg, 1980

Apronopa van Achterberg, 1980: 75; Tobias 1986: 195; Fischer 1991: 8; Wharton 1994: 640; Belokobylskij 1998: 169, 217; Belokobylskij and Tobias 2007: 10; Yu et al. 2016.

######### Diagnosis.

Paraclypeal fovea small, far from inner border of eye. Mandibles small, tridentate, without transverse carina. Upper tooth small; median tooth rather wide and short; lower tooth wide, lobe shaped. Antenna thickened; first flagellar segment longer than second segment. Mesoscutum without postero-medial mesoscutal pit; notauli present on horizontal surface of mesoscutum reaching half or two thirds of mesoscutum; precoxal sulcus present, wide or narrow, oblique, crenulate-rugose, rarely almost smooth; propodeum with different types of sculpture, without areas delineated by carinae. Marginal cell of forewing always long; vein r longer than pterostigma width; vein 2-SR present and rather distinctly sclerotized; veins m-cu and cu-a always strongly postfurcal; subvertical vein 2-SR+M long; first subdiscal cell closed postero-apically by vein CU1b; vein CU1a arising from vein 3-CU1 distinctly behind its middle. Metasoma more or less distinctly depressed dorso-ventrally; first metasomal tergite without dorsope; second tergite usually striate-rugulose in basal one-third or two thirds.

######### Hosts.

Unknown.

######## Apronopa haeselbarthi

Taxon classificationAnimaliaHymenopteraBraconidae

van Achterberg, 1980

Figs 1 , 2

Apronopa haeselbarthi van Achterberg, 1980: 75; Tobias 1986: 195; Fischer 1991: 9; Yu et al. 2016.

######### Type material.

Holotype: female, Germany, Dransfeld, B/L 2.vi.1966 (Haeselbarth leg.) (ZISP). Paratypes: 1 female, 1 male, Germany, Schotten, Hessen, Fi., Streu, v.1967 (Haeselbarth leg.) (♀ in RMNH, ♂ in ZISP).

**Description.** Female (holotype).

***Head.*** In dorsal view, 1.6 times as wide as long, 1.2 times as wide as mesoscutum, smooth, with temple rounded behind eyes. Eye in lateral view 1.3 times as high as wide and 1.5 times as wide as temple medially. POL equal to OD; OOL 2.5 times OD. Face 1.9 times as wide as high; inner margins of eyes subparallel. Clypeus 2.5 times as wide as high, slightly concave ventrally. Paraclypeal fovea almost reaching half distance between clypeus and inner border of eye. Mandible almost parallel-sided, about as long as its maximum width. Upper tooth small; middle tooth rather wide and short, weakly directed upwards; lower tooth wide, curved and obtuse. Antenna more than 9-segmented (apical segments missing). Scape 1.8 times as long as pedicel. First flagellar segment 2.2 times as long as its apical width, 1.3 times as long as second segment. Second flagellar segment 1.6 times, third to seventh segments 1.3–1.5 times as long as their maximum width.

***Mesosoma.*** In lateral view about as long as high. Mesoscutum (dorsal view) about as long as its maximum width, smooth, sparsely setose. Notauli present on horizontal surface of mesoscutum reaching anterior two-third of mesoscutum. Prescutellar depression smooth, with median and lateral carinae, 2.8 times as long as its maximum width. Precoxal sulcus wide, smooth, not reaching anterior and posterior margins of mesopleuron. Posterior mesopleural furrow weakly crenulate. Propodeum sculptured, with several small smooth areas. Propodeal spiracles very small, its diameter 0.2 times as large as distance from spiracle to anterior margin of propodeum.

***Wings.*** Length of forewing 2.4 times its maximum width. Marginal cell 4.2 times as long as its maximum width, ending almost on apex of wing. Vein 3-SR 1.5 times as long as vein 2-SR. Vein SR1 2.8 times as long as vein 3-SR. First subdiscal cell 3.0 times as long as its maximum width. Hindwing 4.3 times as long as its maximum width.

***Legs.*** Hind femur 4.0 times as long as its maximum width. Hind tibia weakly widening towards apex, 8.5 times as long as its maximum subapical width, about as long as hind tarsus. First segment of hind tarsus 1.8 times as long as second segment.

***Metasoma.*** First tergite weakly and curvedly widening towards apex, 1.1 times as long as its apical width, rugose-reticulate. Second tergite distinctly striate with reticulation in two-thirds baso-medially. Ovipositor 3.6 times as long as first tergite, almost as long as metasoma, 2.2 times as long as hind femur.

***Colour.*** Body, flagellar segments of antenna and pterostigma dark brown to black. Mandible and legs brown. First metasomal tergite similar in colour to second and third tergites. Wings almost hyaline.

***Length.*** Body 2.5 mm, forewing 3.0 mm, hindwing 2.2 mm.

**Figure 1.**
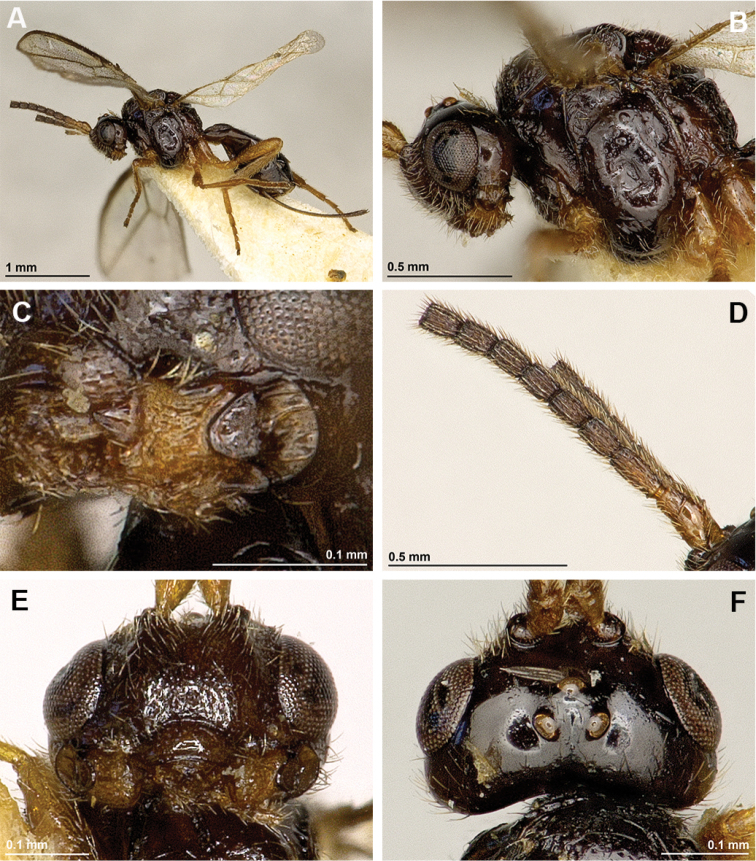
*Apronopahaeselbarthi* van Achterberg, 1980 (female, holotype) **A** Habitus, lateral view **B** Head and mesosoma, lateral view **C** Mandible **D** Antenna **E** Face, frontal view **F** Head, dorsal view.

**Figure 2.**
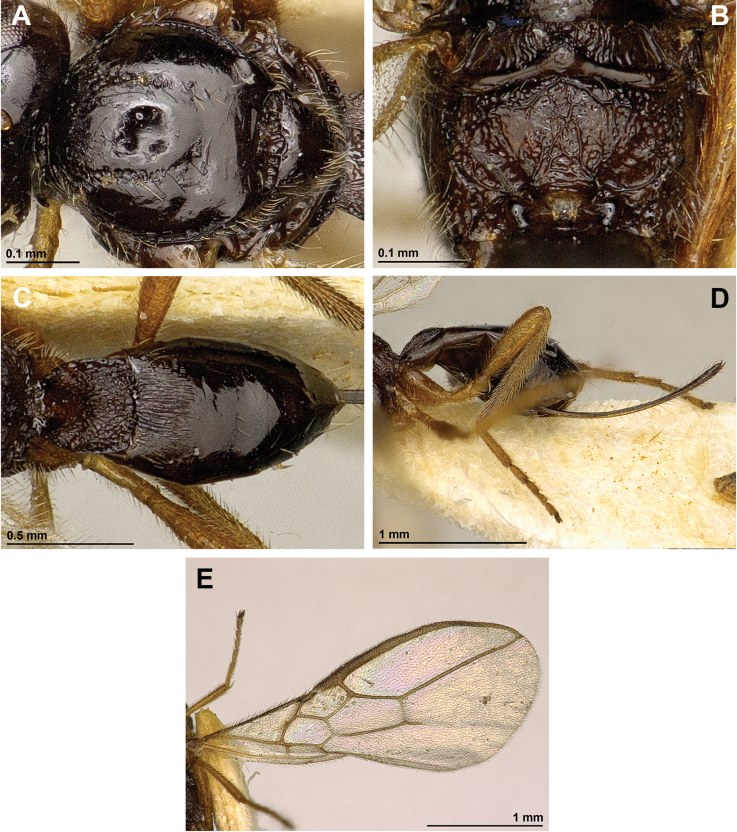
*Apronopahaeselbarthi* van Achterberg, 1980 (female, holotype) **A** Mesonotum, dorsal view **B** Propodeum, dorsal view **C** Metasoma, dorsal view **D** Hind leg, metasoma and ovipositor, lateral view **E** Forewing.

######### Variation.

Length of body 2.0–2.5 mm, forewing 2.0–3.0 mm. Antennae with 23 segments.

***Male.*** Body length 1.9 mm. Forewing length 2.4 mm. Otherwise similar to female.

######### Comparative diagnosis.

This species is similar to *A.ussuricola* Belokobylskij, 1998 (Russia), but differs from it in having the eye in lateral view 1.5 times as wide as temple medially (1.2 times in *A.ussuricola*), face 1.9 times as wide as high, distinctly and densely punctate-rugulose (1.6 times and weakly and sparsely punctate-rugulose in *A.ussuricola*), clypeus 2.5 times as wide as high (3.0 times in *A.ussuricola*), vein 3-SR 1.5 times as long as vein 2-SR (2.0 times in *A.ussuricola*), and ovipositor 3.6 times as long as first metasomal tergite (2.1 times in *A.ussuricola*).

######### Distribution.

Germany, Slovakia.

######## Apronopa levis

Taxon classificationAnimaliaHymenopteraBraconidae

Papp, 2007

Figs 3 , 4

Apronopa levis Papp, 2007: 15; Yu et al. 2016.

######### Type material.

Holotype: female, North Korea, Prov. Kangwon Kumgang-san, 24.ix.1978, in the woods around Oe-Kungang resthouse, No. 396 (A. Vojnits & L. Zombori leg.), Type No. 10970 (HNHM).

######### Description.

Female (holotype).

***Head.*** In dorsal view, 1.8 times as wide as long, 1.4 times as wide as mesoscutum, smooth, with temple rounded behind eyes. Eye in lateral view 1.3 times as high as wide and 1.1 times as wide as temple medially. POL 0.8 times OD; OOL 3.1 times OD. Face 1.8 times as wide as high; inner margins of eyes subparallel. Clypeus 2.5 times as wide as high, slightly concave ventrally. Paraclypeal fovea not reaching middle of distance between clypeus and inner border of eye. Mandible almost parallel-sided, about as long as its maximum width. Upper tooth small, wide and obtuse; middle tooth rather wide and short, directed forward; lower tooth wide, curved and obtuse. Antenna more than 20-segmented (apical segments missing). Scape 1.5 times as long as pedicel. First flagellar segment 2.5 times as long as its apical width, 1.4 times as long as second segment. Second flagellar segment 1.5 times, third to 18^th^ segments 1.3–1.4 times as long as their maximum width.

***Mesosoma.*** In lateral view 1.1 times as long as high. Mesoscutum (dorsal view) 0.9 times as long as its maximum width. Notauli present, on horizontal surface of mesoscutum reaching almost half of mesoscutum. Prescutellar depression smooth, with median and lateral carinae, 2.5 times as long as its maximum width. Precoxal sulcus narrow, crenulate, reaching anterior margin of mesopleuron but not reaching posterior margins of mesopleuron. Posterior mesopleural furrow crenulate. Propodeum anteriorly densely rugose, posteriorly (on its long declivous part) almost smooth, with several wrinkles. Propodeal spiracles small, its diameter 0.2 times as large as distance from spiracle to anterior margin of propodeum.

***Wings.*** Length of forewing 2.6 times its maximum width. Marginal cell 4.4 times as long as its maximum width, ending on apex of wing. Vein 3-SR 1.8 times as long as vein 2-SR. Vein SR1 2.4 times as long as vein 3-SR. First subdiscal cell 2.5 times as long as its maximum width. Hind wing 5.3 times as long as its maximum width.

***Legs.*** Hind femur 4.0 times as long as its maximum width. Hind tibia weakly widened at apex, 9.0 times as long as its maximum subapical width, about as long as hind tarsus. First segment of hind tarsus 1.9 times as long as second segment.

***Metasoma.*** First tergite weakly curvedly, widened at apex, about as long as its apical width, reticulate-rugose. Second tergite entirely smooth. Ovipositor 1.8 times as long as first tergite, 0.7 times as long as metasoma, 3.0 times as long as hind femur.

***Colour.*** Body, flagellar segments of antenna and pterostigma brown to dark brown. Mandible, scape, pedicel and legs light brown. First metasomal tergite similar in colour to second and third tergites. Wings almost hyaline.

***Length.*** Body 3.0 mm, forewing 3.1 mm, hindwing 2.1 mm.

***Male.*** Unknown.

**Figure 3.**
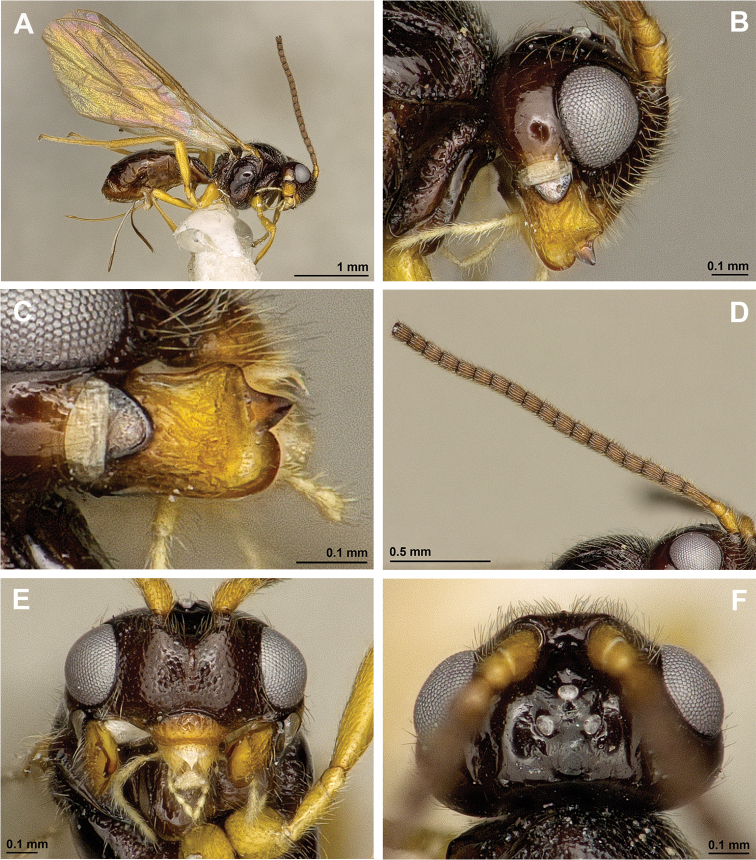
*Apronopalevis* Papp, 2007 (female, holotype) **A** Habitus, lateral view **B** Head, lateral view **C** Mandible **D** Antenna **E** Face, frontal view **F** Head, dorsal view.

**Figure 4.**
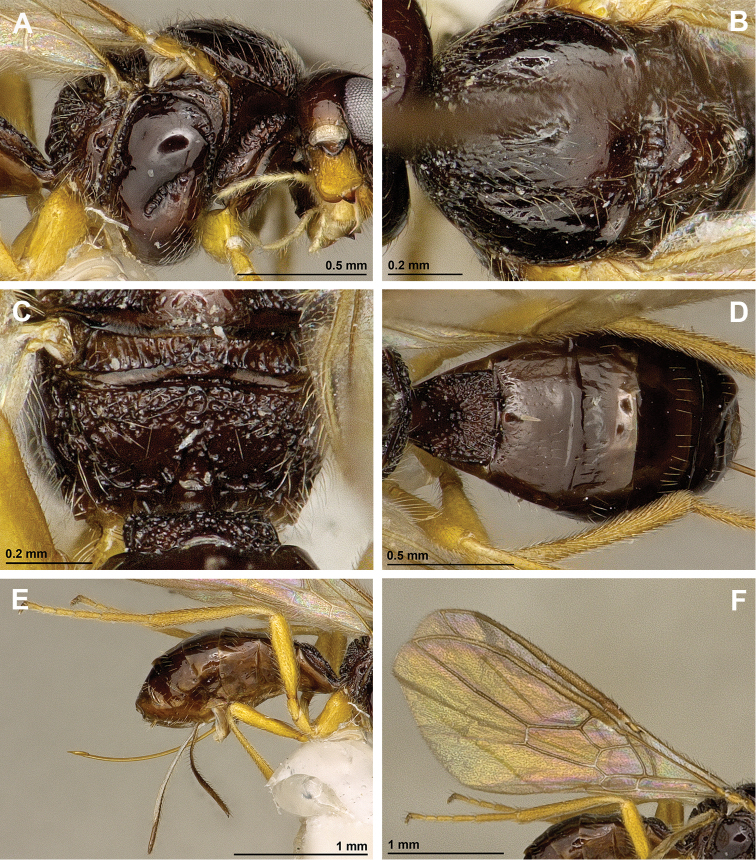
*Apronopalevis* Papp, 2007 (female, holotype) **A** Mesosoma, lateral view **B** Mesonotum, dorsal view **C** Propodeum, dorsal view **D** Metasoma, dorsal view **E** Hind leg, metasoma and ovipositor, lateral view **F** Forewing.

######### Comparative diagnosis.

This species is very similar to *Apronopaussuricola* Belokoblyskij, 1998 (Russian Far East) but differs from them in having an entirely smooth second tergite (tergite striate with rugosity baso-medially in *A.ussuricola*), clypeus 2.5 times as wide as high (3.0 times in *A.ussuricola*), first flagellar segment 2.5 times as long as its maximum width (2.0 times in *A.ussuricola*), and ovipositor 1.8 times as long as first metasomal tergite (2.1 times in *A.ussuricola*).

######### Distribution.

North Korea

######## Apronopa ussuricola

Taxon classificationAnimaliaHymenopteraBraconidae

Belokobylskij, 1998

Figs 5 , 6

Apronopa ussuricola Belokobylskij, 1998: 217; Yu et al. 2016.

######### Type material.

Holotype: female, Primorskiy kray, env. Ussuriysk, dry meadow, 15.vi.1993 (S. Belokobylskij leg.) (ZISP).

######### Description.

Female (holotype).

***Head.*** In dorsal view, 1.8 times as wide as long, 1.4 times as wide as mesoscutum, smooth, with temple rounded behind eyes. Eye in lateral view 1.4 times as high as wide and 1.2 times as wide as temple medially. POL equal to OD; OOL 3.0 times OD. Face 1.6 times as wide as high; inner margins of eyes subparallel. Clypeus 3.0 times as wide as high, slightly concave ventrally. Paraclypeal fovea not reaching middle distance between clypeus and inner border of eye. Mandible almost parallel-sided, as long as its maximum width. Upper tooth very small; middle tooth rather wider and short, directed forwards; lower tooth wide, curved and obtuse. Antenna more than 27-segmented (apical segments missing). Scape 1.1 times as long as pedicel. First flagellar segment 2.0 times as long as its apical width, 1.3 times as long as second segment. Second flagellar segment 1.3 times as long as its maximum width, third to 25^th^ segments 1.2–1.4 times as long as their maximum width.

***Mesosoma.*** In lateral view 1.1 times as long as high. Mesoscutum (dorsal view) 0.9 times as long as its maximum width. Notauli present on horizontal surface of mesoscutum reaching two-thirds of mesoscutum. Prescutellar depression smooth, with median and lateral carinae, about 2.0 times as long as its maximum width. Precoxal sulcus rather wide, crenulate, not reaching anterior and posterior margins of mesopleuron. Posterior mesopleural furrow shortly crenulate. Propodeum rugose-reticulate, with smooth latero-medial areas. Propodeal spiracles rather small, its diameter 0.4 times as large as distance from spiracle to anterior margin of propodeum.

***Wings.*** Length of forewing 2.2 times its maximum width. Marginal cell 4.5 times as long as its maximum width, ending on apex of wing. Vein 3-SR 2.0 times as long as vein 2-SR. Vein SR1 2.6 times as long as vein 3-SR. First subdiscal cell 2.5 times as long as its maximum width. Hindwing 5.0 times as long as its maximum width.

***Legs.*** Hind femur 4.0 times as long as its maximum width. Hind tibia weakly widened at apex, 7.8 times as long as its maximum subapical width, about as long as hind tarsus. First segment of hind tarsus 1.7 times as long as second segment.

***Metasoma.*** First tergite weakly widening towards apex, about as long as its apical width, rugose-reticulate. Second tergite in baso-medial half striate with reticulation. Ovipositor 2.1 times as long as first tergite, 1.1 times as long as metasoma, 3.4 times as long as hind femur.

***Colour.*** Body, flagellar segments of antenna and pterostigma brown to dark brown. Mandible, scape, pedicel and legs light brown. First metasomal tergite similar in colour to second and third tergites. Wings almost hyaline.

***Length.*** Body 2.0 mm, forewing 2.4 mm, hindwing 1.5 mm.

***Male.*** Unknown.

**Figure 5.**
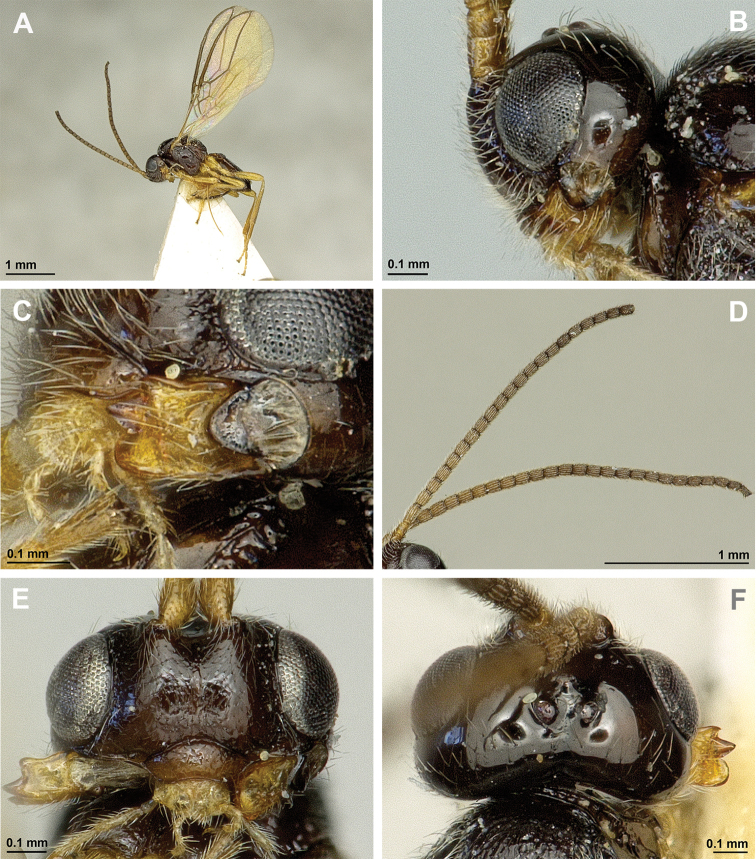
*Apronopaussuricola* Belokobylskij, 1998 (female, holotype) **A** Habitus, lateral view **B** Head, lateral view **C** Mandible **D** Antenna **E** Face, frontal view **F** Head, dorsal view.

**Figure 6.**
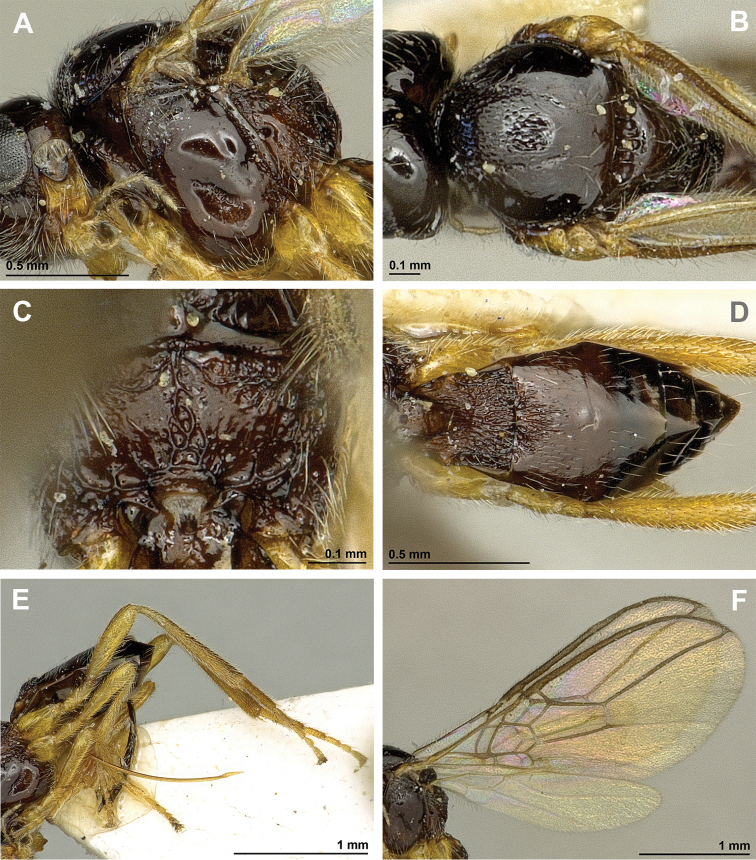
*Apronopaussuricola* Belokobylskij, 1998 (female, holotype) **A** Mesosoma, lateral view **B** Mesonotum, dorsal view **C** Propodeum, dorsal view **D** Metasoma, dorsal view **E** Hind leg, metasoma and ovipositor, lateral view **F** Fore- and hindwings.

######### Comparative diagnosis.

This species is similar to *Apronopahaeselbarthi* van Achterberg, 1980 (Slovakia and Germany); differences between both species are showed under the description of the latter species.

######### Distribution.

Russia (south of Far East).

####### Key to *Apronopa* species

**Table:** 

1	Second metasomal tergite entirely smooth (Fig. 4D). Body length 3.0 mm. North Korea	***A.levis* Papp (♀)**
–	Second metasomal tergite striate with rugosity in baso-medial one- or two-thirds (Figs 2C, 6D). Body length 1.9–2.5 mm	**2**
2	Eye in lateral view 1.5 times as wide as temple medially (Fig. 1B). Face 1.9 times as wide as high, distinctly and densely punctate-rugulose (Fig. 1E).Vein 3-SR 1.5 times as long as vein 2-SR (Fig. 2E). Ovipositor 3.6 times as long as first metasomal tergite (Fig. 2D). Body length 1.9–2.5 mm. Germany, Slovakia	***A.haeselbarthi* van Achterberg (♀♂)**
–	Eye in lateral view 1.2 times as wide as temple medially (Fig. 5B). Face 1.6 times as wide as high, weakly and sparsely punctate-rugulose (Fig. 5E).Vein 3-SR 2.0 times as long as vein 2-SR (Fig. 6F). Ovipositor about 2.0 times as long as first metasomal tergite (Fig. 6E). Body length 2.0 mm. Russia (Far East)	***A.ussuricola* Belokobylskij (♀)**

## Discussion

Most of the genera belonging to the *Aspilota* group (Peris-Felipo et al. 2014) are rather monomorphic in the structures of metasoma, which are usually laterally compressed, entirely smooth behind the first tergite, and the first tergite always with a distinct dorsope. The only exception is the genus *Apronopa* van Achterberg, 1980, which was recently described from Europe and placed within the *Aspilota* generic-group - and compared with the very large genus *Aspilota* Foerster, 1863 (van Achterberg 1980). However, this genus is characterized by the combination of unique and previously unknown characters (in this generic group) such as the presence of dorso-ventrally depressed metasoma, the absence of dorsope on the first metasomal tergite, and usually the presence of sculpturing on the base of the second metasomal tergite. Only three species have been described so far in this rare genus, *A.haeselbarthi* known only from Germany and Slovakia (van Achterberg 1980, Capek and Lukas 1989) and two species from the Eastern Palaearctic, *A.ussuricola* from the south of the Russian Far East (Belokobylskij 1998) and *A.levis* from North Korea (Papp 2007). The first two species have distinct sculpturing on the second tergite (one of the main features of *Apronopa*), but the North Korean species is characterized by an entirely smooth second tergite; however, other characters indicate that it belongs to the genus *Apronopa*. The lack of sculpturing on the second tergite in *A.levis* additionally underlines its position in the *Aspilota* generic-group. Furthermore, Wharton (1994) mentioned that several specimens from undescribed species were seen in the Colorado Museum and that this material would be described by T. Munk; however, that material has not been described yet.

An additional aspect to highlight is the rarity of specimens in collections, which hinders a better understanding (including molecular characters) of this peculiar genus. Any new samples and study of their phylogenetic position would allow us to better understand its position within the *Aspilota* group.

## Supplementary Material

XML Treatment for Apronopa

XML Treatment for Apronopa haeselbarthi

XML Treatment for Apronopa levis

XML Treatment for Apronopa ussuricola
